# Racial and ethnic differences in predictors of participation in an intergenerational social connectedness intervention for older adults

**DOI:** 10.1186/s12877-024-04679-x

**Published:** 2024-01-17

**Authors:** Omolola E. Adepoju, Chinedum O. Ojinnaka, Jason Pieratt, Jessica Dobbins

**Affiliations:** 1 Humana Integrated Health Systems Sciences Institute, University of Houston, Houston, USA; 2 Tilman J Fertitta Family College of Medicine, Department of Health Systems and Population Health Sciences, University of Houston, Houston, USA; 3 College of Health Solutions, Arizona State University, Phoenix, USA; 4 Humana Inc, Louisville, USA

**Keywords:** Social isolation, Social connectedness, Older adults, Race/ethnicity

## Abstract

**Background:**

Social connectedness is a key determinant of health and interventions have been developed to prevent social isolation in older adults. However, these interventions have historically had a low participation rate amongst minority populations. Given the sustained isolation caused by the COVID-19 pandemic, it is even more important to understand what factors are associated with an individual’s decision to participate in a social intervention. To achieve this, we used machine learning techniques to model the racial and ethnic differences in participation in social connectedness interventions.

**Methods:**

Data were obtained from a social connectedness intervention that paired college students with Houston-area community-dwelling older adults (> 65 yo) enrolled in Medicare Advantage plans. Eligible participants were contacted telephonically and asked to complete the 3-item UCLA Loneliness Scale. We used the following machine-learning methods to identify significant predictors of participation in the program: k-nearest neighbors, logistic regression, decision tree, gradient-boosted decision tree, and random forest.

**Results:**

The gradient-boosted decision tree models yielded the best parameters for all race/ethnicity groups (96.1% test accuracy, 0.739 AUROC). Among non-Hispanic White older adults, key features of the predictive model included Functional Comorbidity Index (FCI) score, Medicare prescription risk score, Medicare risk score, and depression and anxiety indicators within the FCI. Among non-Hispanic Black older adults, key features included disability, Medicare prescription risk score, FCI and Medicare risk scores. Among Hispanic older adults, key features included depression, FCI and Medicare risk scores.

**Conclusions:**

These findings offer a substantial opportunity for the design of interventions that maximize engagement among minority groups at greater risk for adverse health outcomes.

## Background

Social connectedness is a key social determinant of health. Often described in terms of social isolation or loneliness, it refers to the interpersonal, physical, and emotional connection that can affect health outcomes [1]. Social isolation is indicative of a lack of social relationships or support and has been defined to encompass structural and functional components [2–4]. Loneliness, on the other hand, is a subjective assessment of social isolation that captures the contrast between actual and desired social connectedness [1–5]. Over the past two years, the direct and indirect consequences of the COVID-19 pandemic have worsened loneliness among older adults with a recent study reporting that about 56% among older adults experienced loneliness due to the pandemic [6]. Protective measures such as social distancing and self-isolation, albeit successful in controlling the spread of disease, could have also increased feelings of loneliness among older adults. In addition, COVID-19 morbidity and mortality rates were highest among older adults, contributing to a large loss of family and loved ones.

Given the close association between social isolation, loneliness and chronic conditions, this concerning trend becomes dangerous for older adults, a population where 90% of individuals have one or more chronic diseases [7]. Social isolation and loneliness have been linked with physical risk factors such as increased blood pressure, obesity, and decreased immune system function, and chronic conditions such as heart, lung, cardiovascular disease, hypertension, and stroke [8–10]. Perceived social isolation has also been found to be a major predictor of mental health issues such as depression, anxiety [10], and increased risk of dementia [11]. With 40% of adults with debilitating disabilities or chronic conditions reporting feelings of social isolation and loneliness due to their pain [12], this relationship can trigger a downward spiral that ultimately results in poorer health.

With the increasing population of racially and ethnically diverse older adults in the U.S, [13] it is important to recognize the disparities in social isolation rates, chronic conditions, and worsened healthcare outcomes for racialized groups. Earlier work suggests older Black and Hispanic adults have significantly fewer ‘discussion’ partners and smaller networks [14]. This, paired with the fact that minority older adults are disproportionately affected by social problems such as inadequate income, insufficient access to resources, and degraded neighborhood and community environments, places them at greater risk for social isolation compared to their white counterparts [15]. This disparity is important considering the association between social networks and the health and well-being of older adults [16]. Chronic diseases are 1.5-2 times more prevalent in racial minorities, with the greatest differences found in diabetes and cancer [17–19]. Worse self-reported health ratings have also been associated with minority groups, particularly Black and Hispanic patients, who respectively had 13.8% and 10% poor health ratings compared to their White counterparts at 8.3%. Mortality rates for Blacks are also higher due to heart disease, strokes and most cancer types [20–22]. While greater structural barriers to health care services among Black and Hispanic individuals, such as insurance coverage and cost, may be contributing to these significant differences, there is a growing body of evidence pointing to the involvement of community and environmental factors [23, 24]. These factors include social connectedness or isolation, and the resulting loneliness, as a driver of chronic illness progression and poor outcomes [25].

Over the past few years, many social connectedness interventions have been developed and studied for their effectiveness [26]. These interventions are generally divided into three categories: personal contact encounters, community-based activities, and mobile technological encounters. Personal contact encounters are defined by scheduled in-person meetups, whether it be one-on-one encounters with students or support groups. For example, the intergenerational “Time after Time” program paired older adults with younger students for regular meetings, and found that 95.5% of adult participants reported feeling more connected to their community, and 89.7% reported the program as a positive contribution to their emotional well-being [27]. Intergenerational programs are a known method for improving social connectedness [28] and empowerment in older adults [29], and have been adopted in a variety of contexts and participants including university students [30, 31].

Community-based activities are designed with the objective of promoting social connection through various activities, such as volunteering or physical exercise. For example, the “Lively Lads” program, which organized exercise classes for older adult males, found programs like these were the most effective and engaging when connecting older adults of similar gender and interests [32]. Mobile technology-based interventions have grown during the pandemic, and typically connect older adults with family and professionals through regular virtual meetings. The Apple-Tree video-call intervention program, is an example of such intervention. The program led vulnerable older adults through facilitated educational seminars, was able to sustain a high participation (83.3%) rate and high goal-achievement rates [33].

Social connectedness interventions have generally shown protective effects, regardless of intervention type or sub-population. However, a fundamental gap among these interventions is the low participation rate among minority groups, as previous literature notes that only 4.5% of studies focused on non-white participants, compared to 41% of studies having over 60% white participants [34]. Other notable efforts have reported low participation rates from racialized groups [35]. Positive impacts have been demonstrated when racial or ethnic communities are targeted by culturally competent interventions to improve social connectedness. In one such study, where at-risk, urban older adults were recruited to participate in diverse group activities ranging from educational workshops to yoga over a 6-month period, significant improvements in self-reported loneliness were observed: from 82% of participants experiencing moderate loneliness to 48.3% reporting not feeling lonely post-intervention [36]. In addition to promoting health equity, this suggests culturally tailored projects can contribute to better outcomes. Hence, there is a need to elucidate factors associated with disparate racial and ethnic groups and their participation in social connectedness interventions. Using machine learning techniques, this study modeled racial and ethnic differences in predictors of participation in intergenerational social connectedness interventions for older adults.

## Methods

### Program and data

Data were obtained from a national health insurance provider and consisted of administrative data for persons meeting the following inclusion criteria: (1) membership in the insurer’s Medicare Advantage (MA) plans, (2) residence in Houston, Texas at the time of the program launch, (3) aged 65 years or older at the time of the launch, between May 2020-November 2020.

Eligible older adults were invited to participate in a telephonic intergenerational linkage program (ILP) to address social connectedness. University student participants were trained to offer companionship by virtually engaging with their assigned older adult at least once a week.

Program enrollment was offered in English, however participating Spanish-speaking students were preferentially paired with Spanish-speaking older adults. The ILP was designed in response to (1) the unintended effect of social distancing on older adults who were already at risk for social isolation and (2) the cancellation of several in-person field training opportunities for health professions students, during the early phase of the pandemic. Additional details about the ILP and its enrollment process were captured in an earlier study [37]. Ethical approval for this study was granted by the Institutional Review Board of the University of Houston in November 2020 (IRB ID: STUDY00002617).

### Measures

The primary outcome was a binary measure indicating participation in the ILP, which was defined as program enrollment and > 1 visit among all eligible beneficiaries. Predictor variables included demographic characteristics, Medicare enrollment, composite risk index scores, comorbidities, and healthcare utilization. Demographic characteristics included individual level factors such as age, gender, race/ethnicity, primary language, disability status, low-income status (i.e., Medicare beneficiaries with income below 150% of the poverty threshold). Medicare enrollment information included coverage length in months, Medicare/Medicaid dual enrollment, and primary care provider (PCP) attribution type. Risk scores included Charlson comorbidity index (CCI)^32^, Functional Comorbidity Index (FCI)^33^, Medicare risk score, and Medicare prescription risk score. Comorbid conditions were identified through CCI and FCI scores. Measures of healthcare utilization included emergency department (ED) visits, inpatient (IP) admissions, and IP days. The inclusion of non- individual factors is important because the deployment of social connectedness interventions is often made at a non-individual level.

### Model development

We used supervised machine-learning methods to predict ILP participation. Because they do not rely on traditional regression model assumptions, machine learning approaches are typically effective in handling large amounts of explanatory variables in situations when an exploratory approach is needed, such as that presented in this study. In addition, ML is appealing because collinearity does not matter, allowing investigators to examine multiple individual and organizational factors at once. The working data were split into (1) a training dataset (75% of the data), and (2) a testing dataset (25% of the data). The training data were used to predict intervention participation using the variables mentioned in the measures section.

Five supervised machine-learning methods were implemented to predict participation by eligible subjects in the social connectedness intervention: (1) k-nearest neighbors (k-NN), (2) logistic regression, and (3) decision tree and ensembles of decision trees, including (4) gradient-boosted decision tree and (5) random forest. These five models were selected based on their previously shown strong discriminative performance in prediction-related classification problems [38, 39]. We applied the five-machine learning (ML) algorithms to our data stratified by the following race/ethnicity categories: non-Hispanic White, non-Hispanic Black, and Hispanic.

#### Analytic approach

The predictive performance of each model was evaluated using predictive accuracy (%) and the area-under-the-receiver-operating-characteristic (AUROC) curve. Both approaches are commonly used evaluation metrics [38, 39]. While accuracy represents the proportion of the total number of predictions that were correctly classified, AUROC measures the diagnostic ability of a binary classification model, The prediction models were built using the best-fitting parameters for each model, which were obtained by using the ‘GridSearchCV’ command with five-fold cross-validation in Python. ‘GridSearchCV’ is an approach that exhaustively considers all combinations of parameters to perform hyperparameter tuning of models.

To identify the contribution of each considered predictor to the models, the feature importance of the tree models (i.e., gradient-boosted decision tree, and random forest) was computed. All analyses were performed in Python (v3.9.0; Beaverton, OR).

## Results

Table 1 shows the demographic characteristics of eligible beneficiaries by race/ethnicity for the full sample. We observed racial and ethnic differences by age, such that younger participants were more likely to be non-Hispanic Black, or Hispanic (35% non-Hispanic White vs. 48% non-Hispanic Black vs.53% Hispanic; *p* < 0.001). While approximately 56% of the sample were female, the proportion varied by race/ethnicity, with 55% non-Hispanic White, 60% non-Hispanic Black and 52% Hispanic (*p* = 0.01). Significant differences were also observed for primary language, with 80% of Hispanic participants reporting limited or poor English proficiency, compared to 14% non-Hispanic Whites and 1% non-Hispanic Black (*p* < 0.001). Among non-Hispanic Whites, 7% had dual enrollment in Medicaid and Medicare, while non-Hispanic Blacks and Hispanic participants respectively had 17% and 20% dual enrollees (*p* < 0.001). Disability status was more common among non-Hispanic Blacks with 18% reporting disability, compared to 10% among non-Hispanic Whites and 8% among Hispanic participants (*p* < 0.001). The proportion of eligible participants who identified as low-income was almost two times greater among non-Hispanic Blacks, and three times greater among Hispanic participants, when compared to the proportion of non-Hispanic Whites (*p* < 0.001). Finally, the level of ILP participation also varied across race and ethnicity, with participation rates at 3% among non-Hispanic Whites, 5% among non-Hispanic Blacks and 4% among Hispanic participants (*p* < 0.001). CCI score was 4.31 among non-Hispanic Whites, 4.38 among non-Hispanic Blacks and 3.94 among Hispanic participants (*p* = 0.03). Pre-index IP admissions did not vary by race.

**Table 1:** Demographic characteristics by race/ethnicity (*N* = 17,750)

	Total (*n*=17,750)		Non-Hispanic White (*n*=11,412)		Non-Hispanic Black (*n*=4501)		Hispanic (*n*=1838)		p-value
	n	(%)	n	(%)	n	(%)	n	(%)	
**Age**									<0.001
65–69	7137	(40.2)	3979	(34.9)	2179	(48.4)	979	(53.3)	
70–74	5274	(29.7)	3510	(30.8)	1266	(28.1)	498	(27.1)	
75–79	3089	(17.4)	2264	(19.8)	606	(13.5)	219	(11.9)	
> 80	2250	(12.7)	1659	(14.5)	450	(10.0)	141	(7.70)	
**Gender**									<0.001
Male	7861	(44.2)	5179	(45.4)	1808	(40.2)	874	(47.6)	
Female	9889	(55.7)	6233	(54.6)	2693	(59.8)	963	(52.4)	
**Primary language**									<0.001
Not English	3109	(17.5)	1578	(13.8)	59	(1.30)	1472	(80.1)	
English	14,641	(82.5)	9834	(86.2)	4442	(98.7)	365	(19.9)	
**Medicare/Medicaid Dual Enrollment**									<0.001
Not dual-enrolled	15,836	(89.2)	10,616	(93.0)	3750	(83.3)	1470	(80.0)	
Dual-enrolled	1914	(10.8)	796	(7.0)	751	(16.7)	367	(20.0)	
**Disability Status**									<0.001
Not living with a disability	15,691	(88.4)	10,298	(90.2)	3703	(82.2)	1690	(92.0)	
Living with disability	2059	(11.6)	1114	(9.8)	798	(17.7)	147	(8.0)	
**Low-Income Status**									<0.001
Non-low-income	14,611	(82.3)	10,080	(88.3)	3317	(73.7)	1214	(66.1)	
Low-income	3139	(17.7)	1332	(11.7)	1184	(26.3)	623	(33.9)	
**Social Connectedness Intervention**									0.001
Did not participate	17,072	(96.2)	11,041	(96.8)	4262	(94.7)	1769	(96.3)	
Participated	678	(3.8)	371	(3.3)	239	(5.3)	68	(3.7)	
**Charlson Comorbidity Index, mean (SD)**	4.27	(0.01)	4.31	(0.02)	4.38	(0.03)	3.94	(0.04)	0.03
**Pre-index Inpatient Admits/1000, mean (SD)**	98.1	(3.43)	98.7	(4.33)	98.3	(6.8)	89.7	(11.5)	0.45

Column percentages sum up to 100%

### Prediction of intervention participation by race/ethnicity

The predictive abilities of the models, represented by the test accuracy (%) and AUROC, are shown in Table 2. When stratified by race/ethnicity, test accuracy rates were comparable, but AUROC values varied slightly. For non-Hispanic White beneficiaries, the k-NN model with its best parameters yielded a test accuracy of 97.0% and an AUROC of 0.691. The logistic regression model with its best parameters yielded a test accuracy of 97.0% and an AUROC of 0.738. The decision tree model with its best parameters yielded a test accuracy of 97.0% and an AUROC of 0.721. The gradient-boosted decision tree model with its best parameters yielded a test accuracy of 96.6% and an AUROC of 0.759. The random forest model with its best parameters yielded a test accuracy of 96.6% and an AUROC of 0.748.

**Table 2:** Predictive performance of each model, measured by test accuracy (%) and AUROC, stratified by race/ethnicity

	Test Accuracy (%)				AUROC			
	All	White	Black	Hispanic	All	White	Black	Hispanic
k-NN	96.0	97.0	95.0	96.0	0.612	0.691	0.661	0.621
Logistic regression	96.0	97.0	95.0	97.0	0.730	0.738	0.683	0.791
Decision tree	96.1	97.0	95.0	97.0	0.647	0.721	0.661	0.709
Gradient-boosted decision tree	96.1	96.6	95.0	96.8	0.739	0.759	0.701	0.838
Random forest	96.1	96.6	95.1	96.8	0.740	0.748	0.644	0.744

For non-Hispanic Black beneficiaries, the k-NN model with its best parameters yielded a test accuracy of 95.0% and an AUROC of 0.661. The logistic regression model with its best parameters yielded a test accuracy of 95.0% and an AUROC of 0.683. The decision tree model with its best parameters yielded a test accuracy of 95.0% and an AUROC of 0.661. The gradient-boosted decision tree model with its best parameters yielded a test accuracy of 95.0% and an AUROC of 0.701. The random forest model with its best parameters yielded a test accuracy of 95.1% and an AUROC of 0.644.

For Hispanic beneficiaries, the k-NN model with its best parameters yielded a test accuracy of 96.0% and an AUROC of 0.621. The logistic regression model with its best parameters yielded a test accuracy of 97.0% and an AUROC of 0.791. The decision tree model with its best parameters yielded a test accuracy of 97.0% and an AUROC of 0.709. The gradient-boosted decision tree model with its best parameters yielded a test accuracy of 96.8% and an AUROC of 0.838. The random forest model with its best parameters yielded a test accuracy of 96.8% and an AUROC of 0.744.

### Feature importance by race/ethnicity

**Fig. 1:**
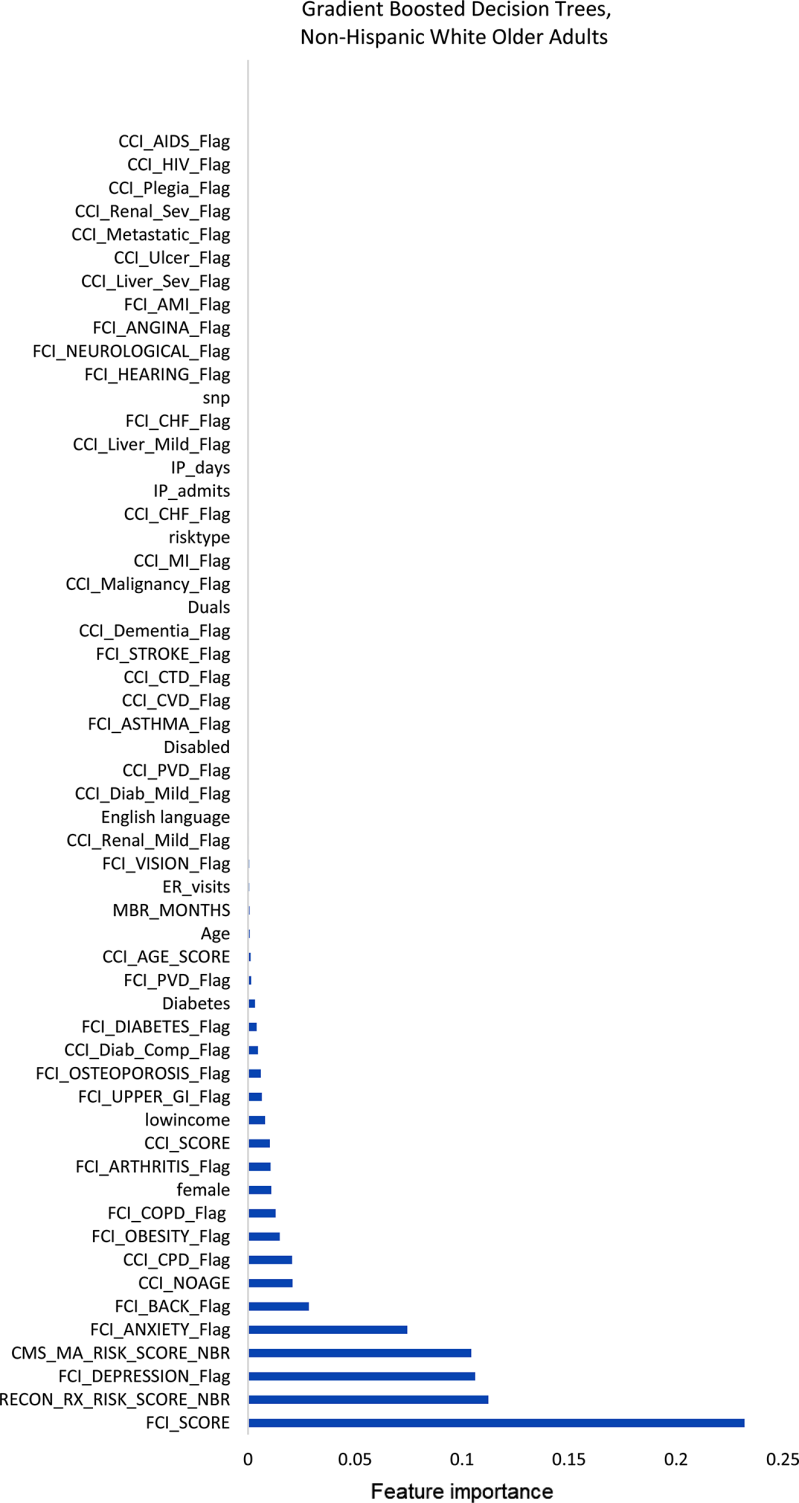
Feature importance plot predicting social-connectedness intervention participation in non-Hispanic White older adults. AIDS, acquired immune deficiency syndrome; AMI, acute myocardial infarction; CCI, Charlson Comorbidity Index; CHF, congestive heart failure; COPD, chronic obstructive pulmonary disease; CTD, connective tissue disease; CVD, cardiovascular disease; Diab, diabetes; ER, emergency department; FCI, functional comorbidity index; GI, HIV, human immunodeficiency virus; IP, inpatient; MI, myocardial infarction; PCP, primary care provider; PVD, peripheral vascular disease; Rx, pharmacy; Sev, severe

**Fig. 2:**
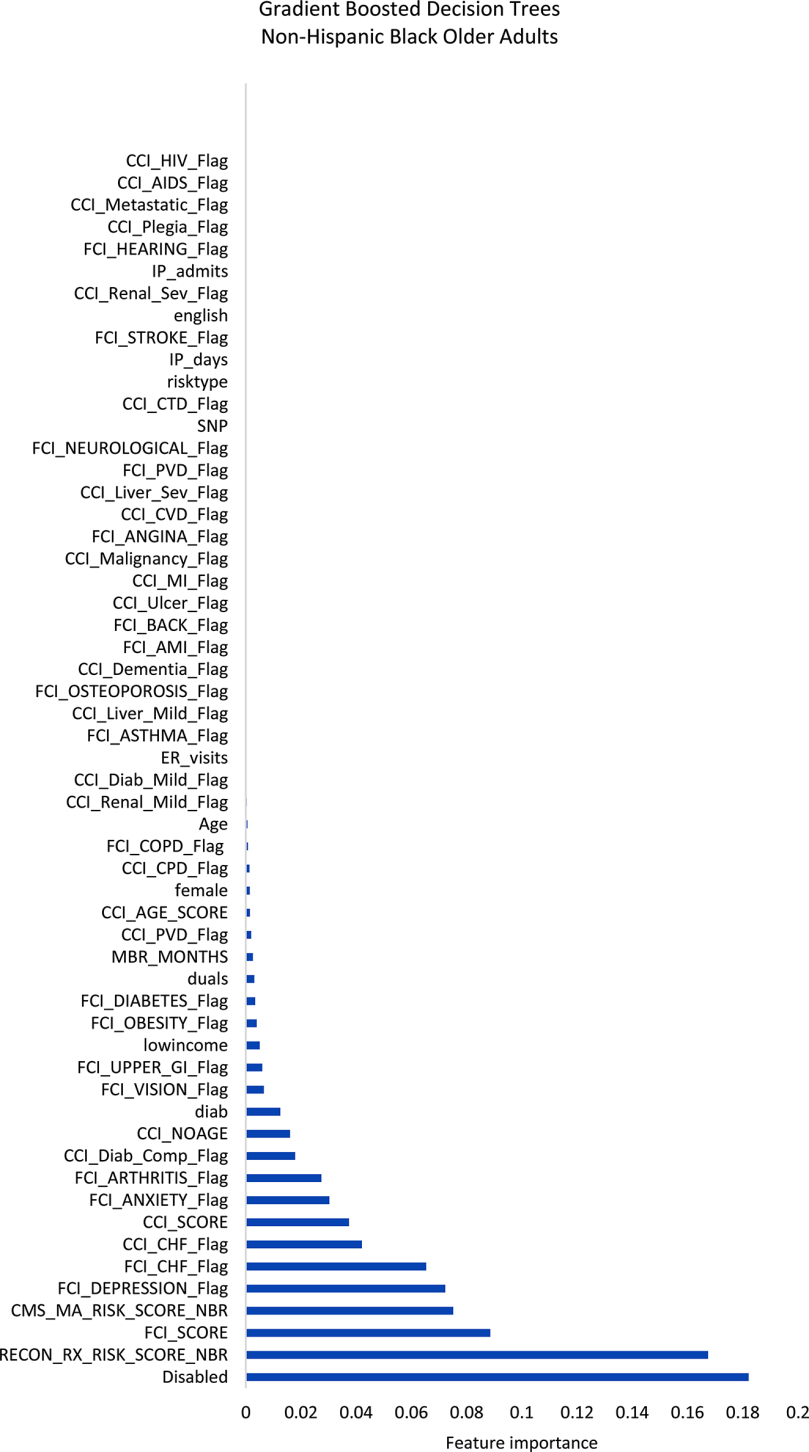
Feature importance plot predicting social-connectedness intervention participation in non-Hispanic Black older adults. AIDS, acquired immune deficiency syndrome; AMI, acute myocardial infarction; CCI, Charlson Comorbidity Index; CHF, congestive heart failure; COPD, chronic obstructive pulmonary disease; CTD, connective tissue disease; CVD, cardiovascular disease; Diab, diabetes; ER, emergency department; FCI, functional comorbidity index; GI, HIV, human immunodeficiency virus; IP, inpatient; MI, myocardial infarction; PCP, primary care provider; PVD, peripheral vascular disease; Rx, pharmacy; Sev, severe

**Fig. 3:**
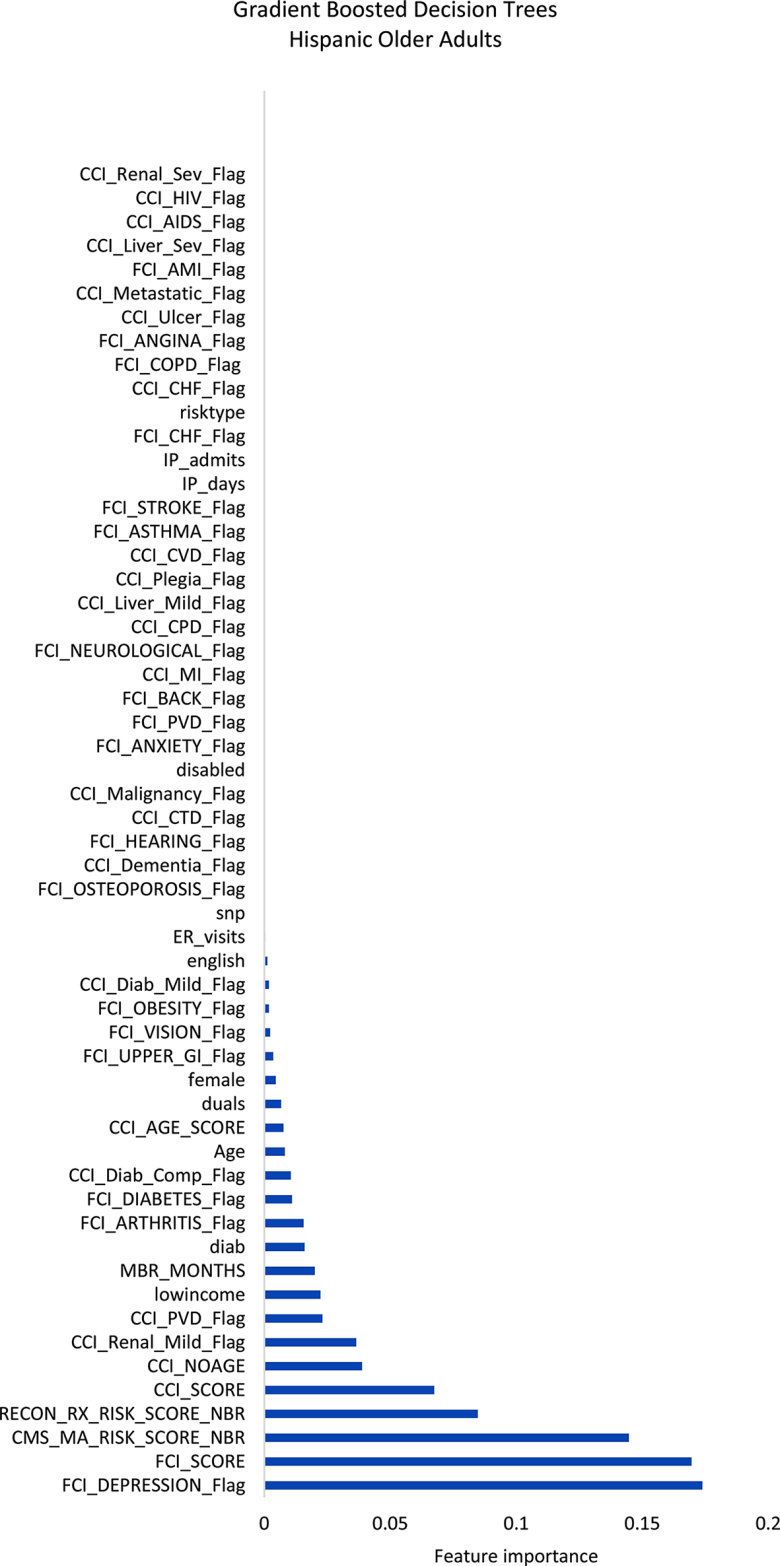
Feature importance plot predicting social-connectedness intervention participation in Hispanic older adults. AIDS, acquired immune deficiency syndrome; AMI, acute myocardial infarction; CCI, Charlson Comorbidity Index; CHF, congestive heart failure; COPD, chronic obstructive pulmonary disease; CTD, connective tissue disease; CVD, cardiovascular disease; Diab, diabetes; ER, emergency department; FCI, functional comorbidity index; GI, HIV, human immunodeficiency virus; IP, inpatient; MI, myocardial infarction; PCP, primary care provider; PVD, peripheral vascular disease; Rx, pharmacy; Sev, severe

Figures 1–3 display the feature importance in the gradient-boosted decision tree model.

Among non-Hispanic White older adults (Fig. 1), the gradient-boosted decision tree models identified the most important features to be the FCI score, followed distantly by the Medicare prescription risk score, the Medicare risk score, and the depression and anxiety indicators within the FCI. Among non-Hispanic Black older adults (Fig. 2), the same model identified the most important features of those patients with a disability, Medicare prescription risk score, followed distantly by the FCI score and Medicare risk score. Among Hispanic older adults (Fig. 3), the model identified the most important features to be depression, followed closely by the FCI scores and Medicare risk scores.

## Discussion

In this study, we used machine learning algorithms to examine racial and ethnic differences in predictors of participation in a social connectedness intervention for Medicare-insured older adults who had reported social isolation. While participation by non-Hispanic White MA patients was best predicted based on traditional risk score profiles, the contribution of depression, and to a slightly lower degree, anxiety, in the model is notable. Most notably, are the prominence of disability status for predicting participation among Black older adults, and the lead role of depression in predicting participation in Hispanic older adults. Not surprisingly, the composite risk scores like FCI were always near the top in terms of importance, albeit less so in non-Hispanic Black older adults. Our study findings fill a critical gap by highlighting the difference in determinants of participation in a social connectedness intervention across race and ethnicity. These findings suggest there might be utility in race/ethnicity-specific recruitment approach for social connectedness interventions. The difference in determinants of program participation also suggests a need for more studies that explore barriers and facilitators of participation in social connectedness interventions.

Disability status as a predictive feature of ILP participation in non-Hispanic Black older adults represents a unique target for program designers or managers. Of course, there is considerable research to show that individuals living with disabilities are more likely to experience social isolation and to report feelings of loneliness. This, combined with the lack of mobility and perhaps increased time spent in the home, may have contributed to the increased participation based on this lived experience. On the other hand, depression as the lead feature predicting ILP participation in Hispanic and a secondary predictor in non-Hispanic White older adults appears to be a novel finding. Social stigma related to reporting mental health symptoms like depression (or anxiety) are still more prevalent in older age strata and these concerns have been shown to vary by both race and ethnicity in the past. Given the rapid growth in the past couple of years of social isolation, loneliness, and mental health concerns like depression, the relationship of these factors on program engagement bears further investigation.

Multiple studies have demonstrated the positive impact of social connectedness on health outcomes such as chronic disease prevalence and disease management. Of note, a meta-analysis reported a strong social relationship was associated with a 50% reduction in mortality risk [1]. Although this study operationalized social relationships as a composite measure comprising distinct concepts such as loneliness and social isolation, the findings highlight the need for interventions aimed at improving social connectedness. The realization of the importance of social isolation as a determinant of health has led to interventions that aim to increase social connectedness among older adults [34]. The intervention modality often varies with some, like the ILP in the index study, requiring active involvement by participants and others not involving active involvement [3, 34]. The intervention outcomes have also varied with some reporting a significant improvement in social connectedness and others reporting no significant impact [3, 34]. The inconsistent impact of these interventions on social connectedness suggests a need to understand factors that might affect the impact of social connectedness interventions on target outcomes. For such studies to be generalizable, there will be a need for a targeted recruitment approach that increases the likelihood of equitable participation in these interventions across race/ethnicity.

Considering that social connectedness interventions are often deployed at the organizational level, several organizational considerations can impact efforts to address social isolation in older adults. For example, health plans should promote policy design with inclusive coverage for social support services, and community engagement programs. Organizations can also support the use of user-friendly technologies that enable older adults to access information, connect with healthcare professionals, and participate in virtual social activities. Finally, organizations can establish partnerships with community organizations, senior centers, and non-profits that specialize in addressing social isolation, as these collaborative efforts can enhance the reach and impact of interventions.

### Public health implications

The importance of this work lies in the potential ability to tailor or target interventions for older, minority individuals and communities.

Targeted interventions to increase participation in social connectedness interventions by eligible non-White older adults are critical considering that racial and ethnic disparities in health outcomes remain ubiquitous in the United States. For example, Non-Hispanic Blacks continue to have the highest mortality rates compared to other racial groups in the United States [40]. Considering the significant association between social connectedness and mortality, targeted interventions to improve participation in social connectedness among older adults who feel socially isolated offer a unique opportunity to address race/ethnic-related disparities in morbidity and mortality.

### Limitations

The chosen analytic approach, involving multiple ML-classification methods and highly-efficient optimization of parameters, offers a robust set of results for guiding the implementation of future social connectedness interventions. However, these findings are based on MA eligible participants in a single, large, urban metropolitan area. Generalizability of these findings to other parts of the country, globe, or even to rural areas may require replication studies. We also did not examine strata of the MA-eligible population identifying in racial groups outside of White or Black, nor ethnic designations beyond the dichotomous Hispanic and non-Hispanic designations. Finally, this analysis refrained from delving into examination of the role of intersectionality between race and ethnicity or any other demographics. Future research is needed to investigate all of these extant questions.

As we acknowledge in the introduction, a number of structural, environmental, social and psychosocial factors may confound the relationship between social connection and health. The burden of these factors is not evenly or fairly distributed throughout the population. The way that these factors interact and influence such a dynamic relationship is also likely to vary across different racial or ethnic community lines, and even neighborhoods. In particular, the role of insurance coverage and costs are also likely to play a modifying role in the relationship of social connectedness and health outcomes (perceived or otherwise). However, this study was restricted to similar groups of older adults all enrolled in the same MA plan under a single private insurance provider eliminating some of that population- and meso-level variability.

## Conclusion

Social connectedness is a key social determinant of health, especially among vulnerable populations. The features associated with social connectedness program participation differ by racial and ethnic demographics. Program recruitment efforts that account for these differences and associations could increase more equitable participation across race/ethnicity groups and have the potential to reduce related health disparities.

## Data Availability

Data used in this analysis were acquired from a payer partner, and we do not have permission for data sharing. Inquiries regarding this data can be made to this email: oadepoju@central.uh.edu.

## Abbreviations

AUROC curve: area-under-the-receiver-operation-characteristic curve
CCI: Charlson comorbidity index
ED: emergency department
FN: false negatives
FP: false positives
FCI: Functional Comorbidity Index
IP: inpatient
ILP: intergenerational linkage program
k-NN: k-nearest neighbors
ML: machine learning
MA: Medicare Advantage
PCP: primary care provider
ROC: receiver operating characteristic
SNP: special needs plan
TN: true negative
TP: true positive
